# Efficacy of a Combined Intramedullary, Periarticular Injection, and Intraarticular Tranexamic Acid Application on Postoperative Bleeding in Total Knee Arthroplasty: A Retrospective Case-Matched Study

**DOI:** 10.1155/2022/9175189

**Published:** 2022-12-10

**Authors:** Theerawit Hongnaparak, Khanin Iamthanaporn, Pakjai Tuntarattanapong, Varah Yuenyongviwat

**Affiliations:** Department of Orthopedics, Faculty of Medicine, Prince of Songkla University, Songkhla 90110, Thailand

## Abstract

**Background:**

Topical tranexamic acid (TXA) has been widely used to reduce postoperative blood loss following total knee replacement (TKA). This study aimed to evaluate the effect of combined intramedullary, periarticular injection, and intraarticular TXA application in patients who underwent TKA as compared to those who did not.

**Methods:**

This was a retrospective case-matched study of 111 patients. We evaluated the transfusion rate and postoperative drainage of 56 patients who underwent TKA using combined topical tranexamic acid application (directly pushed into the femoral intramedullary canal and tibia base, with periarticular soft tissue injection and injected into the knee cavity via a drain tube) and the control group of 55 patients.

**Results:**

In the control and TXA groups, 7.14% and 1.81% of the patients received blood transfusions, respectively (*p*=0.176). The closed-suction drainage output at 0–8 h and total drainage output were significantly lower in the TXA group than those in the control group (*p* < 0.001).

**Conclusion:**

Application of topical TXA with the combined method (intramedullary, periarticular injection, and intraarticular) in TKA decreases postoperative suction drainage and may reduce the need for postoperative blood transfusion.

## 1. Background

Reducing postoperative bleeding after total knee replacement (TKA) can improve postoperative outcomes and decrease complications [1, 2]. According to previous reports, the average rate of blood transfusion in patients who had TKA was 44% [3]. However, the complications of blood transfusion after TKA include an increased deep infection rate, allergic reaction, transfusion reactions, and blood-borne infection [4]. Several studies have investigated methods to reduce postoperative blood loss following TKA such as hypotensive anesthesia, using a tourniquet, topical hemostatic agents, postoperative limb positioning, and tranexamic acid (TXA) [5].

TXA application is one of the standard methods that is widely used currently [6, 7]. TXA is an indirect fibrinolytic inhibitor that can be applied via several routes, including intravenous [8, 9], oral, topically applied [10], or combination routes [11]. Topical TXA involves direct application to the surgical site. Differences in the application method have been reported, including pouring TXA solution into the knee joint cavity and removal with a sucker [12], injecting TXA into the knee joint after wound closure [13, 14], and periarticular TXA injection [15].

The intramedullary femoral cutting guide is generally used in conventional TKA. This instrument penetrates the intramedullary canal from the distal femur [16], which causes significant blood loss due to injury of the endosteal tissue and increased blood loss following the operation [17, 18]. In our practice, we use topical TXA with a novel application method. We first apply TXA directly into the femoral intramedullary canal and tibia base before prosthesis implant, followed by periarticular injection, and finally, TXA is applied to the knee cavity via a drain tube after wound closure. No previous study evaluated the efficacy of intramedullary TXA application on postoperative bleeding in TKA.

This study aimed to evaluate the effect of topical application of tranexamic acid with the combined method (intramedullary, periarticular injection, and intraarticular) in reducing postoperative bleeding in TKA. We hypothesized that this combined application method would reduce postoperative bleeding in TKA.

## 2. Material and Methods

### 2.1. Patients

This was a retrospective study conducted at a tertiary hospital from January 2016 to April 2021. Patients' data were extracted from the hospital's electronic database. This study was approved by the local ethics committee and Institutional Review Board. The need for patient consent was waived by the ethics committee.

We enrolled patients who underwent primary TKA by a single surgeon. Patients with incomplete data were excluded. The inclusion criteria included patients who had unilateral TKA for primary OA. The exclusion criteria included patients who had previous knee surgery and TKA combined with another procedure.

Patients who underwent TKA between January 2016 and October 2019 were designated as the control group (patients who did not receive TXA). Patients who underwent TKA between October 2019 to April 2021 and received local TXA application with the combined method were designated as the experimental group.

### 2.2. TKA Procedure

All TKA procedures were performed by a single surgeon with a uniform surgical technique. The medial parapatellar approach with lateral patellar subluxation was used in all the cases. A cemented posteriorly stabilized knee prosthesis was used, and the patellar was resurfaced. (NexGen legacy posterior-stabilized (LPS-flex), Zimmer, Warsaw, Indiana) The distal femoral cut was performed with the intramedullary guide and the proximal tibial cut with the extramedullary guide. A pneumatic tourniquet was inflated throughout the operation.

### 2.3. Combined TXA Administration Procedure

Ten milliliters (500 mg) of TXA was directly pushed into the femoral intramedullary canal and tibia base, while 10 ml (500 mg) of TXA was injected into the area of the quadriceps muscle tendon, medial, and lateral capsule before the prostheses were implanted. Finally, 10 ml (500 mg) of TXA were injected into the knee cavity via a drain tube after wound closure. The drain tube in both groups was clamped for 3 h and then released.

### 2.4. Postoperative Care

All patients received the same postoperative care and rehabilitation protocol. Patients received oral celecoxib at a dose of 200 mg/day for 1 week for postoperative pain control. Ankle pumping and quadricep isometric exercises were started immediately after the operation. Patients were ambulated with a supportive device, and range of motion exercises were initiated a day after the surgery. Drainage outputs were recorded every 8 h, and the drain tube was removed 48 h after the operation. The patients received a blood transfusion if their hematocrit level was less than 30% or if they had symptoms of anemia. Patients were evaluated clinically and monitored for any postoperative complications at 14 days and 6 weeks after the operation.

### 2.5. Statistical Analyses

Patient demographic data such as age, weight, height, body mass index (BMI), preoperative hematocrit, preoperative hemoglobin level, platelet count, operative times, hospital stay, and postoperative drainage were compared between the groups with an independent *t*-test. Chi-square test was used to evaluate the effects of sex, American Society of Anesthesiologists (ASA) classification, transfusion rates, and postoperative complications. All statistical analyses were conducted in R, version 3.1.0 (R Foundation for Statistical Computing, Vienna, Austria). Statistical significance was defined as *p* ≤ 0.05.

## 3. Results

A total of 111 patients who met the study criteria were included in the study. Fifty-five patients received local TXA, while the remaining 56 were in the control group. The demographic data of the patients are summarized in Table 1. There were no demographic differences between the two groups. Further, no differences were found between the groups in terms of sex, age, side, weight, height, BMI, ASA classification, preoperative hemoglobin level, preoperative hematocrit, or platelet count.

Blood transfusions were administered to 7.1% (4/56) of the patients in the control group and 1.8% (1/55) of the patients in the TXA group. However, this difference was not statistically significant (*p*=0.176). Conversely, the total drainage output and the closed suction drainage output at 0–8 h were significantly different between the TXA group and the control group (*p* ≤ 0.001 and <0.001, respectively), but they were not significant at 8–16 h, 16–24 h, and 24–48 h (*p*=0.419, 0.075 and 0.298, respectively, Table 2).

The operative time was lower in the control group than that in the TXA group (148.8 ± 23 minutes and 163.2 ± 29.6 minutes, respectively, *p*=0.005). There was no difference in hospital stay between the groups (3.8 ± 1.3 days in the control group and 3.6 ± 1.1 days in the TXA group, *p*=0.383). Further, there was no incidence of deep infection, deep vein thrombosis, pulmonary embolism, or wound hematoma requiring reoperation in any of the cases.

## 4. Discussion

There is limited evidence on the role of adding TXA directly into the femoral intramedullary canal and tibia base before the prosthesis implant, combined with periarticular injection, and adding it into the knee cavity via a drain tube after wound closure. This study was conducted to evaluate the effectiveness of the combined method in decreasing blood loss in patients with TKA as compared to the control group. Overall, we found that this combined method could reduce postoperative drainage. Further, it demonstrated a nonsignificant trend towards reducing the blood transfusion rate.

This study found that patients in the TXA group had a lower closed suction drainage output as compared to patients in the control group. This result was the same as that observed in previous studies that investigated local TXA. Şahin et al. reported a prospective randomized study that compared patients who were administered 2 g TXA intraarticularly following patellar tendon repair and before wound closure to a control group [19]. Their results showed that patients in the intraarticular TXA group had lower 24 h drainage in comparison to the control group. Another prospective randomized study by Roy et al. compared patients who were administered 5 ml of 500 mg TXA via a drain tube to patients who received 5 ml of 0.9% normal saline and demonstrated that patients in the TXA group had a significant reduction in the drain output 48 h postoperatively [20].

In terms of blood transfusion, our study found the patients in the control group had a 3.94-fold higher blood transfusion rate than the TXA group. However, there was no statistically significant difference between the groups. Our results agree with those of the study by Roy et al., which found that the control group received a six-fold higher blood transfusion rate than the TXA group but that a statistically significant difference between the groups was not observed [20]. Our study and that of Roy et al. both investigated a small cohort (111 patients and 50 patients, respectively); it is likely the small number of patients resulted in low power to demonstrate the differences in blood transfusion.

This study has some limitations. First, this was a retrospective study; therefore, the surgeon and evaluator were not blinded. However, all surgeries were performed by a single surgeon with an identical protocol. Moreover, the outcome of this study was objective and was not significantly affected by evaluator bias. Second, this study had a small number of patients. Thus, it was underpowered to demonstrate the difference in blood transfusion (Power = 0.269, Alpha 0.05). Moreover, while patients who received topical TXA demonstrated a tendency towards a lower blood transfusion rate, this difference was not statistically significant. Further studies with a larger number of participants and a longer duration of data collection would be beneficial to validate these results and confirm whether there is a significant difference in blood transfusion rates. Third, this study did not report the hidden and calculated blood losses due to the limitation of retrospective data. We suggest that this data be reported in further studies. Finally, this study used a liberal transfusion strategy that was a historical rule proposed for patients with poor anesthesia risk (30% of hematocrit) [21]. However, nowadays a restrictive transfusion threshold (hemoglobin concentration of 7 g/dl) is widely used, and a report by Hébert et al. confirmed that this threshold was as effective as the liberal transfusion strategy [22].

## 5. Conclusions

In conclusion, we found that the administration of topical TXA with the combined method that included intramedullary, periarticular injection, and intraarticular administration could decrease postoperative suction drainage in TKA without increasing the complication rate. However, this method did not reduce the transfusion rate. Further studies comparing this combined method with other single local TXA regimens are required to validate these results.

## Figures and Tables

**Table 1:** Demographic data.

Characteristic	Group 1 (control)	Group 2 (TXA group)	*p* value
	*N* = 56	*N* = 55	
Sex (male : female)	6 : 50	8 : 47	0.543
Age (years)	68.3 ± 6.4^*∗*^	69.1 ± 7.7^*∗*^	0.568
Side (left : right)	22 : 34	30 : 25	0.107
Weight (kg)	65.3 ± 10.8^*∗*^	66.4 ± 9.8^*∗*^	0.565
Height (cm)	153 ± 6.1^*∗*^	154.4 ± 7.1^*∗*^	0.266
BMI (kg/m^2^)	27.9 ± 4^*∗*^	27.8 ± 3.6^*∗*^	0.973
ASA classification (II : III)	47 : 9	38 : 17	0.065
Preop hemoglobin (g/dL)	13.3 ± 0.7^*∗*^	13 ± 1.4^*∗*^	0.155
Preop hematocrit (%)	40.1 ± 2.7^*∗*^	39.2 ± 4.4^*∗*^	0.203
Platelet count (×10^3^/*μ*L)	261 ± 52.5^*∗*^	273.5 ± 49.9^*∗*^	0.203

^*∗*^Values are expressed as mean ± SD.

**Table 2:** Closed-suction drainage output.

Characteristic	Control group	TXA group	*p* value
	*N* = 56	*N* = 55	
0–8 h	412.3 ± 202.2^*∗*^	151.2 ± 87.8^*∗*^	<0.001
8–16 h	27.1 ± 55.9^*∗*^	19.4 ± 44.5^*∗*^	0.419
16–24 h	72.7 ± 46.2	88.6 ± 46.8	0.075
24–48 h	142.9 ± 78.3	160.6 ± 99.7	0.298
Total drainage	690.7 ± 308.5	458.5 ± 207.9	<0.001

^*∗*^Values are expressed as mean ± SD.

## Data Availability

The datasets generated during this current study are available from the corresponding author upon reasonable request.

## Abbreviations

TKA: Total knee replacement
TXA: Tranexamic acid
BMI: Body mass index
ASA: American Society of Anesthesiologists.
